# Epidemiology of human respiratory tract infection in Chongqing, China after COVID-19–based on surveillance data encompassing 17 respiratory pathogens

**DOI:** 10.3389/fcimb.2025.1567341

**Published:** 2025-06-23

**Authors:** Tingting Li, Jiang Long, Zhourong Li, Yu Xiong, Luzhao Feng, Mingyue Jiang, Yanxia Sun, Li Qi

**Affiliations:** ^1^ Chongqing Center for Disease Control and Prevention (Chongqing Academy of Preventive Medicine), Chongqing, China; ^2^ Chongqing Disease Prevention and Public Health Research Center Construction Program, Chongqing, China; ^3^ School of Public Health, Chongqing Medical University, Chongqing, China; ^4^ School of Population Medicine and Public Health, Chinese Academy of Medical Sciences & Peking Union Medical College, Beijing, China

**Keywords:** respiratory tract infection, epidemiology, multiple respiratory pathogens, SARS-CoV-2, surveillance

## Abstract

**Background:**

Respiratory tract infections (RTIs) are an important public health concern, and SARS-CoV-2 affects the epidemic pattern of RTIs globally. National multipathogenic surveillance for RTIs was conducted in mid-October 2023. However, baseline data on the pathogen spectrum of RTIs in Chongqing, China, before mid-October 2023 are limited.

**Methods:**

A descriptive analysis was conducted to determine the epidemiology of RTIs in Chongqing, China, in 2023.

Results A total of 1,894 individuals were included in 2023, with an overall positivity rate of 28.7%. The highest overall positivity rate was observed in May 2023 (53.0%). Different predominant respiratory pathogens were observed among different age groups. Among all single-infected individuals, SARS-CoV-2 accounted for 32.1%, followed by IFV, which accounted for 28.2%. In the children group, RSV ranked first, accounting for 15.7%. Among school-aged children, IFV ranked the top, accounting for 46.7%. SARS-CoV-2 ranked the top among adults and the elderly, accounting for 45.5% and 47.0%, respectively.

**Conclusions:**

The local pathogen spectrum of RTIs one year after the onset of COVID-19 showed that SARS-CoV-2 was steady, and viral infection might be the main cause of RTIs. Both upper respiratory tract infections and lower respiratory tract infections (LRIs) showed high RTI positivity rates. The pathogen spectra of upper respiratory tract infections (URIs) and LRIs differ in adults. Holistic surveillance of RTIs is necessary to estimate the local disease burden. Vaccination against respiratory infections remains an important strategy to prevent and control RTIs.

## Introduction

Respiratory tract infections (RTIs) are major global health problems that can be classified into upper respiratory tract infections (URIs) and lower respiratory tract infections (LRIs). LRIs are the fourth leading cause of death worldwide, accounting for 2.50 million deaths, and pose a significant global health challenge (Collaborators GL, 2022). Most RTIs are self-limiting but can lead to severe outcomes including hospitalization and death, especially in immunocompromised young children and the elderly (Echavarría et al., 2018). During the global COVID-19 pandemic, the epidemiology of respiratory pathogens such as influenza, mycoplasma pneumoniae (M. pneumonia) (Meyer Sauteur et al., 2022), and respiratory syncytial virus (RSV) (Chuang et al., 2023), appears to have changed dramatically, showing an obvious downward trend (Li et al., 2022; Yoshioka et al., 2023). The phenomenon could be attributed to the public health and social measures (PHSMs) that were implemented to curb the spread of SARS-CoV-2, such as social distancing, school closures, and mask mandates. However, studies conducted at home and abroad showed that these respiratory pathogens resurged as PHSMs lifted (Mott et al., 2021; Ujiie et al., 2021; Li et al., 2022).

The PHSMs implemented during the COVID-19 pandemic, while effective in containing SARS-CoV-2 transmission, also modulated the incidence of other respiratory infections (Shi et al., 2022; Yuan et al., 2022; Boehm et al., 2023). In late 2022 after Chinese government has shifted COVID-19 prevention and control policies, the long-term impact of pandemic-era measures on the resurgence patterns of other respiratory pathogens remained unclear (Li et al., 2022). Chongqing is the largest subtropical municipality in China, with a heavy burden of respiratory diseases (Qi et al., 2020a; Qi et al., 2020b; Li et al., 2023). Owing to COVID-19 pandemic, the activity of mutations in respiratory pathogens shifted significantly. Follow the WHO declaration of the pandemic’s end in May 2023, PHSMs were gradually relaxed globally. In this context, evaluating the evolving spectrum of respiratory pathogens and monitoring their activity has become critical. Such assessments help guide localized prevention and control policies in response to shifting epidemiological trends.

Following the COVID-19 pandemic, the government realized it was essential to re-evaluate the activity of respiratory pathogens. This could help understand the updated circulation of these pathogens and their impact on the population. As a result, the government support to conduct multipathogen surveillance across most regions of China. Chongqing, an important city in southwestern China, is a key focus. These data will serve as a reference for southern China and even other parts of the country. The previous historical surveillance data of respiratory diseases in China, primarily focused on influenza (National surveillance protocol for influenza (version 2010): China National Influenza Center, 2010), underscore the significance of expanding research to encompass a broader spectrum of other respiratory pathogens. Meanwhile, there were limited data before the national multipathogenic surveillance for RTIs was conducted.

Therefore, our study first reported and analyzed the epidemiology of RTIs with seventeen respiratory pathogens in Chongqing, China, with the goal of revealing the dynamic epidemic characteristics of multiple pathogens after the COVID-19 pandemic (in 2023). Additionally, we aimed to identify the risk groups for severe disease in Chongqing, intending to offer practical references for preventing and controlling respiratory infectious diseases. Through our research, we can establish the baseline of the pathogen spectrum of local respiratory tract infections. Furthermore, it could provide a guidance for the allocation of medical resources (such as medications, hospital beds, medical staff, and vaccines). Beside, these data will serve as a reference for southern China and even other parts of the country.

## Materials and methods

This is a multi-center research led by the Chinese Academy of Medical Sciences to analysis of the characteristics of multi-pathogen composition in cases of acute respiratory infection, with more than 14 joint institutions around China, including Chongqing. National multipathogenic surveillance for RTIs was conducted in mid-October 2023. However, baseline data on the pathogen spectrum of RTIs in Chongqing, China, before mid-October 2023 are limited. Based on the holistic protocol, our sample size was approximately 2000 individuals. According to different economic levels, this cross-sectional study was executed in four districts in Chongqing from 1 January to 31 December 2023, including Wanzhou District, Yongchuan District, Wulong District, and Nan’an District. This study randomly enrolled individuals with acute respiratory infection (Daoud Perez et al., 2022) from four districts in Chongqing, from 12 hospitals (Wulong District People’s Hospital, Fukang Hospital, Wulong District Traditional Chinese Medicine Hospital, Wulong District Maternal and Child Health Hospital, Chongqing No. 5 People’s Hospital, The First Affiliated Hospital of Chongqing Medical and Pharmaceutical College Chongqing Southeast Hospital, The Affiliated People’s Hospital of Three Gorges Medical College, The Affiliated Hospital of Three Gorges Medical College, The Central Health Center of Fenshui Town, The Central Health Center of Longsha Town, and The Affiliated Yongchuan Hospital of Chongqing Medical University).

The criteria of enrollment were as follows: URIs, 1) Individuals of all age groups can be included; 2) Have clinical manifestations of acute infection, including any one of fever (with possible hypothermia considering age), abnormal white blood cells (increase, decrease, or abnormal distribution), and chills; 3) Have any one of the respiratory symptoms simultaneously, including pharyngeal discomfort, dry throat or sore throat, nasal congestion, runny nose, obvious congestion and edema of the nose/pharynx/larynx, cough (new onset or aggravated cough), expectoration, shortness of breath, abnormal auscultation of breath sounds (rales, rhonchi, wheezing, dullness), and chest pain; 4) Sign the informed consent form.

LRIs, 1) Inpatients whose illness onset was within 7 days; 2) Have respiratory symptoms such as fever, cough or sore throat; 3) Chest X - ray (or chest CT) examination shows pneumonia; 4) Sign the informed consent form.

A uniform questionnaire with information regarding demography, diagnosis and treatment, sample collection, and laboratory testing was obtained from all involved individuals. URIs samples (oropharyngeal swabs) were collected from both outpatients and inpatients. Lower respiratory tract samples such as bronchoalveolar lavage fluid, tracheal secretions, or deeply induced sputum are preferred by inpatients; however, oropharyngeal swabs can be collected if lower respiratory tract samples cannot be obtained. Seventeen respiratory pathogens which included SARS-CoV-2, influenza A virus (IFV-A), influenza B virus (IFV-B), RSV, human adenovirus (HAdV), human parainfluenza virus (HPIV) 1-4, human metapneumovirus (HMPV), human coronavirus (HCoV) 229E, NL63, OC43, HKU-1, human rhinovirus (HRV), *M.pneumonia, chlamydia pneumoniae (C. pneumonia)*, were identified by real-time fluorescent polymerase chain reaction testing.

Data analysis was performed by R software (version 4.2) and Microsoft Excel (version 2019; Redmond, WA, USA). Descriptive analysis was employed to illustrate the epidemiological characteristics. Line charts and percentage bar chart were used to show the monthly distribution of positive respiratory pathogens detection and the distribution of respiratory pathogens by age, respectively. Chi-square test was conducted to compare differences between subgroups. P values less (two-tail) than 0.05 were considered statistically significant.

## Results

In total, 2,242 people participated in this research in Chongqing. After data cleaning, a total of 1,894 individuals were included in our study, with an overall positivity rate of 28.7%. The single infection and co-infection rates were 25.8% and 2.9%, respectively. Higher positivity rates were observed among school-aged children, outpatients, and patients with URIs. **Table 1** shows the positive rates of respiratory pathogens in the different groups.

**Table 1:** Positive rates of respiratory pathogens among patients with respiratory infection in Chongqing, in 2023. (N=1,894).

Characteristics	All respiratory pathogens tested			
	No. specimens tested	No. laboratory‐confirmed cases (%)		
		Total	Single infection	Co-infection
Sex				
Male	1,050 (55.4)	290 (27.6)	270 (25.7)	20 (1.9)
Female	844 (44.6)	253 (30.0)	219 (26.0)	34 (4.0)
Age group				
Children (≤5 years)	350 (18.5)	122 (34.9)	102 (29.1)	20 (5.7)
School-aged children (6–17 years)	303 (16.0)	117 (38.6)	105 (34.7)	12 (4.0)
Adults (18–59 years)	628 (33.2)	181 (28.8)	165 (26.3)	16 (2.5)
Older people (≥60 years)	613 (32.4)	123 (20.1)	117 (19.1)	6 (1.0)
Case type				
Inpatients	1,046 (55.2)	195 (18.6)	184 (17.6)	11 (1.0)
Outpatients	848 (44.8)	348 (41.0)	305 (36.0)	43 (5.1)
RTIs type				
URIs	1,438 (75.9)	459 (31.9)	412 (28.7)	47 (3.3)
LRIs	456 (24.1)	84 (18.4)	77 (16.9)	7 (1.5)
Total	1,894 (100.0)	543 (28.7)	489 (25.8)	54 (2.9)

**Figure 1:**
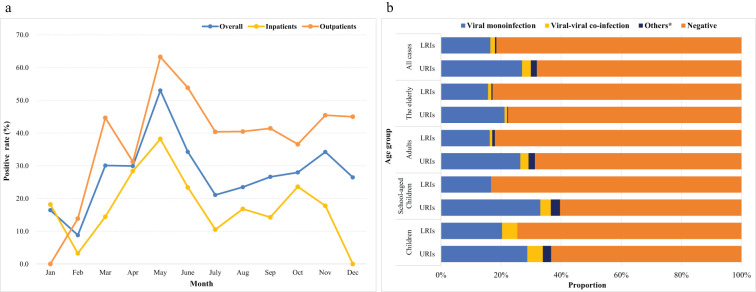
Positive rates **(a)** and respiratory pathogens detection **(b)** in Chongqing, China, in 2023. *Others indicate M. pneumonia, **(C)** pneumonia single infection and they co-infected with other viruses.

The highest overall positivity rate was observed in May 2023 (53.0%), with a higher number of outpatients than inpatients. **Figure 1a** shows the monthly distribution of positive rates among the RTI cases. Different predominant respiratory pathogens were observed among different age groups. The pathogens detection in URIs varied significantly across age groups (χ²=34.79, P < 0.001), whereas no statistically differences were observed in LRIs (χ²=7.54 P=0.581). In the three groups of all cases, the elderly, and the adults, viral monoinfection had a higher rate (27.0%–15.7%), followed by viral-viral coinfection, among both among LRIs and URIs. In the school-aged children group, the proportion bar charts displayed results from limited samples (two samples tested viral monoinfection out of twelve samples). In the children group, viral monoinfection was most frequently identified among the LRIs (20.3%), M. pneumonia, and C. pneumonia were not detected. **Figure 1b** shows the proportion of respiratory pathogens among the different age groups.

**Figure 2:**
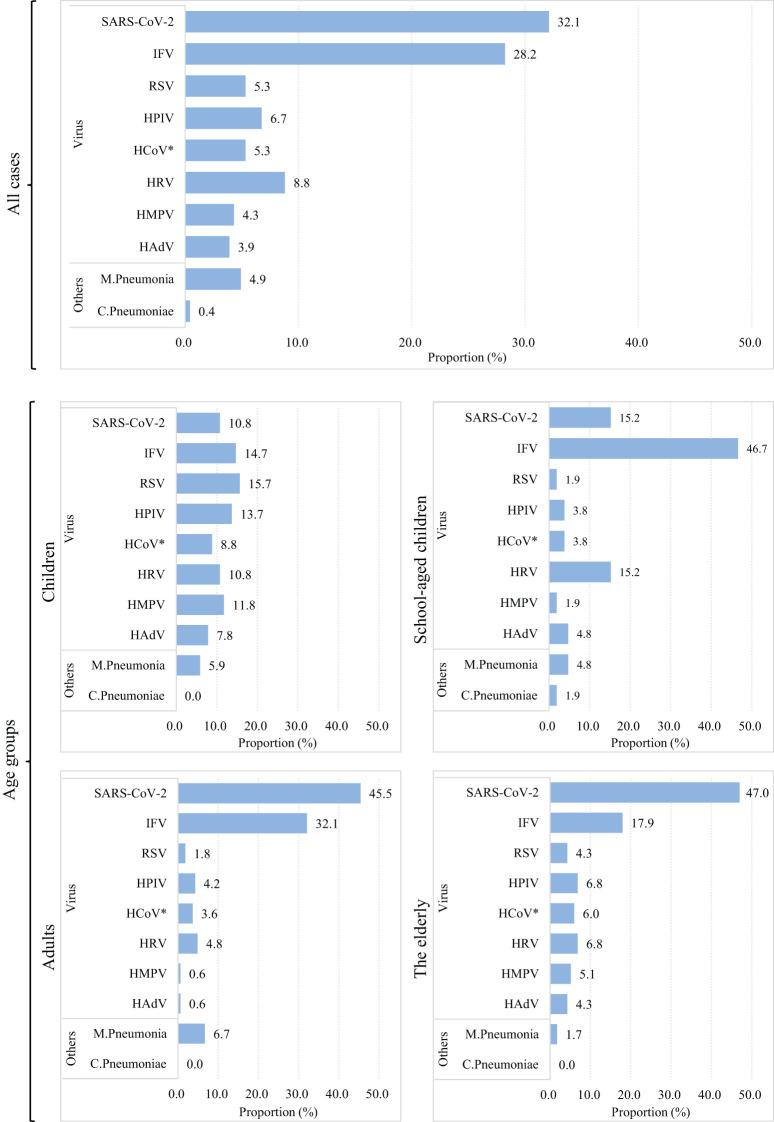
Viral composition of single infection patients in Chongqing China, in 2023. Note: The length of colored bars and the number behind indicate the proportion of each pathogen, calculated by its positive number used as the numerator and the total positive number of all pathogens used as the denominator. *HCoV includs 229E, NL63, OC43, HKU-1.

Among all single-infected individuals, SARS-CoV-2 accounted for 32.1%, followed by IFV, which accounted for 28.2%. In the children group, RSV, IFV, and HPIV ranked the first three, accounting for 15.7%, 14.7%, and 13.7%, respectively. Among school-aged children, IFV ranked the top, accounting for 46.7%, followed by SARS-CoV-2 and HRV, which accounted for 15.2%, respectively. SARS-CoV-2 ranked the top among adults, accounting for 45.5%, followed by IFV (32.1%) (Qi et al., 2020a). SARS-CoV-2 accounted for the majority (47.0%) of the elderly, followed by IFV (17.9%). **Figure 2** illustrates the details of the viral and bacterial compositions of single infections.

## Discussion

Chongqing, a megacity with a larger population than the capital Beijing, holds significant importance in China. Our previous studies have estimated that Chongqing has a substantial influenza- and pneumonia-related mortality burden (Qi et al., 2020a; Qi et al., 2020b; Li et al., 2023). Owing to mutations in respiratory pathogens and immunity debt during the COVID-19 period, people may exhibit increased susceptibility to respiratory pathogens (Zuurbier et al., 2023), which could lead to the recurrence of epidemic peaks of RTIs after the COVID-19 pandemic. Based on our surveillance, the findings revealed that school-aged children, outpatients, and patients with URIs suffered the most respiratory infections, with a higher overall positive rate. Similarly, a community-based cohort study revealed a positivity rate of > 70% among healthy children in New Zealand (Walker et al., 2022). The proportion of people infected with SARS-CoV-2 remains at a relatively high level locally, even PHSMs to curb the spread of SARS-CoV-2 were lifted. This also leading to the relatively low proportion of other pathogens (Oishi et al., 2022; Presti et al., 2024). But in different age groups, the predominant respiratory pathogens were differed; like in children and school-aged children group, the predominant respiratory pathogens were still those prevailed before COVID-19.

We observed that viral monoinfection was prevalent in all cases of respiratory infection in Chongqing, China, in 2023, including both URIs and LTIs. Besides, viral-viral co-infection was also observed in all age groups. Another community-acquired pneumonia study (Liu et al., 2023) conducted from 2009 to 2020 in China indicated that among adults and the elderly, bacterial monoinfection was the leading respiratory pathogen and viral-bacterial co-detection was rare in all age groups. Hence, persistent comprehensive monitoring of respiratory infections was necessary in the local area. Further analysis of the single infection revealed different pathogens among different age groups. In children and school-aged children, IFV, RSV, and HRV were responsible for the foremost respiratory tract infections in 2023. Conversely, in adults and elderly individuals, SARS-CoV-2 was superior to the other seventeen respiratory pathogens. However, before the COVID-19 pandemic, IFV was responsible for most respiratory infections in different age groups in China (Li et al., 2021). Also, our previous study found a resurgence of IFV during COVID-19 in Chongqing, China, implying the high prevalence of IFV locally, even though HCoV remained in a low-level epidemic (Li et al., 2023). There was another report indicating that RSV causes a considerable disease burden for older and high-risk adults (Nguyen-Van-Tam et al., 2022), so attention regarding the older, and adults should not be neglected even though many citations signify children are more vulnerable individuals to RSV (Li et al., 2022).

This study has a few limitations. Our tested pathogens were mostly viruses, common bacteria were not included tested. In addition, the LRI samples from school-aged children were limited compared to other age groups, and more data should be collected for in-depth analysis.

## Conclusions

This descriptive analysis revealed the pathogen spectrum of respiratory infections according to age, URIs, and LRIs. RTIs are an important public health issue, especially in school-aged children. Viral infection may be the main cause of local RTIs, and the prevalence of SARS-CoV-2 remains at a certain level locally. These findings underscore the need to monitor the epidemiological trends in respiratory infections. A comprehensive surveillance of RTIs is necessary to estimate the disease burden, and conducive to implement prevention and control measures such as health advisories for the public, vaccination, and the stockpiling of medical resources, locally.

## Data Availability

The raw data supporting the conclusions of this article will be made available by the corresponding author LQ on reasonable request, without undue reservation.
